# Safety evaluation of subcutaneous and intravenous administration of infliximab: a real-world study based on the FAERS database

**DOI:** 10.3389/fmed.2026.1834815

**Published:** 2026-06-01

**Authors:** Xiaoke Zheng, Minpeng Xie, Huan Luo, Jingyue Cai, Rongrong Xu, Tao Ling

**Affiliations:** 1 Department of Pharmacy, Central People's Hospital of Zhanjiang, Zhanjiang, Guangdong, China; 2 Department of Gastroenterology, Suqian First Hospital, Suqian, Jiangsu, China; 3 Department of Pharmacy, Northern Jiangsu People's Hospital, Yangzhou, Jiangsu, China; 4 Department of Pharmacy, Suqian First Hospital, Suqian, Jiangsu, China

**Keywords:** adverse events, FAERS, infliximab, intravenous, subcutaneous

## Abstract

**Background:**

Inflammatory Bowel Disease (IBD) is characterized by recurrent flare-ups, significantly impairing patients’ long-term quality of life and increasing the risk of complications. As the first chimeric monoclonal antibody targeting tumor necrosis factor-alpha (TNF-α), infliximab blocks TNF-α-mediated inflammatory cascades and has become an important therapeutic agent for moderate-to-severe IBD. Although clinical trials have highlighted its safety and efficacy, real-world data on adverse events (AEs) associated with different administration routes remains limited. This study aims to analyze the U. S. Food and Drug Administration’s Adverse Event Reporting System (FAERS) to compare the spectrum and time-to-onset of infliximab-related AEs across different administration routes, following the READUS-PV guidelines.

**Methods:**

Data from the FAERS database were analyzed using discriminant and stratified analytical methods, and reporting odds ratio (ROR) methods for comparative analysis, and the Weibull distribution for the time to onset of adverse reaction events analysis. The study examined data from the first quarter of 2014 through the first quarter of 2025 to analyze adverse event signals and the time of occurrence between intravenous and subcutaneous infliximab administration.

**Results:**

A total of 178,925 adverse reaction reports associated with infliximab were identified. Among these, 5,144 events were linked to intravenous administration, while 1,084 events were related to subcutaneous administration. The analysis revealed that infection-related adverse reactions were common with both different administration routes of infliximab. Notably, reports of infusion-related reactions were more frequently associated with intravenous administration, while delayed hypersensitivity reactions involving musculoskeletal and connective tissue disorders were more prominently reported in association with subcutaneous administration. Additionally, Weibull distribution analysis indicated a median onset time of 436.5 days for intravenous administration and 376 days for subcutaneous administration. Both routes exhibited early failure signals, suggesting a gradually decreasing risk of AEs over time.

**Conclusion:**

AEs and the time of onset vary across different administration routes of infliximab. Consequently, clinicians should consider these differences when selecting the administration route to balance therapeutic efficacy with the risk of adverse reactions.

## Introduction

Inflammatory Bowel Disease (IBD) refers to diseases related to chronic or remitting/relapsing intestinal inflammation, primarily ulcerative colitis and Crohn’s disease, both of which are common digestive system conditions potentially associated with the interplay of multiple factors such as environmental influences, genetics, infections, and immune dysfunction (1–4). IBD is typically characterized by chronic diarrhea (with or without bleeding), abdominal pain, and weight loss, which significantly reduces patients’ quality of life and even threatens life and health (1, 2). Increased levels of tumor necrosis factor-alpha (TNF-α) has been shown in the serum and mucosa of patients with IBD, and the serum levels are considered to be associated with disease severity (5). As the result, it is suggested that TNF-α is one of the main pro-inflammatory cytokines involved in the pathogenesis of IBD (6).

Infliximab, a chimeric human-murine IgG1 monoclonal antibody against TNF-α, was approved by the U. S. Food and Drug Administration (FDA) for using in adults and pediatric patients aged 6 years and older with moderate-to-severe active Crohn’s disease or ulcerative colitis who have had an inadequate response to conventional therapy, as well as the patient with rheumatoid arthritis, psoriatic arthritis, ankylosing spondylitis, and plaque psoriasis (7–10). Additionally, studies have shown that it is effective in treating perianal fistulas in both adult and pediatric patients with Crohn’s disease (11–13). There was evidence that infliximab may be more effective than purine analogs in inducing and maintaining both clinical remission and mucosal healing in Crohn’s disease (14). Therefore, infliximab has revolutionized the traditional treatment of IBD, especially providing a new option for refractory patients. According to prescribing information, the intravenous dosing regimen of infliximab for IBD is 5 mg/kg at weeks 0, 2, and 6, followed by a maintenance dose every 8 weeks thereafter. For adult patients with moderate to severe active Crohn’s disease who have an inadequate response, the dose may be increased to 10 mg/kg. However, the intravenous infusion administration of infliximab requires most patients to receive treatment in healthcare facilities, which not only inconveniences patients but also consumes additional medical resources (15, 16). To overcome these barriers, subcutaneous infliximab has received FDA approval for the maintenance of treatment of adults with moderate-to-severe active Crohn’s disease or ulcerative (17). Recent studies have proved that there is no difference in efficacy between intravenous and subcutaneous of infliximab, whereas subcutaneous administration of infliximab enhances patient compliance due to its relatively simple and convenient handling (16, 18). Some researches have different conclusions that subcutaneous infliximab maintenance therapy was related to better pharmacokinetic parameters with higher trough concentrations and lower peak concentrations and a stable disease course (19, 20). What’s more, subcutaneous infliximab demonstrated the same or better safety, with the common adverse events (AEs) including injection site reaction (21, 22). In short, subcutaneous or intravenous infliximab for the maintenance therapy of adults with moderate-to-severe active IBD was effective and well-tolerated.

Despite the well-established efficacy and safety of infliximab in clinical trials, these findings are derived from highly selected populations under controlled conditions, which may limit their generalizability to real-world clinical practice. In particular, clinical trials often lack sufficient power to detect rare, delayed, or population-specific AEs, especially in heterogeneous patient populations. In contrast, the U. S. Food and Drug Administration Adverse Event Reporting System (FAERS) provides large-scale real-world pharmacovigilance data, enabling the identification of safety signals across broader populations and over extended periods of drug exposure (23–25). Increasingly, FAERS-based studies have been used to complement clinical trial evidence and support post-marketing safety evaluation. Moreover, the route of administration may influence both pharmacokinetics and immunogenicity, thereby affecting the safety profile of infliximab. These differences are clinically relevant, as they may impact treatment selection, patient adherence, and long-term disease management in IBD.

Therefore, this study aims to systematically compare adverse event profiles and time-to-onset characteristics between intravenous and subcutaneous infliximab using FAERS data, providing real-world evidence to inform clinical decision-making.

## Materials and methods

### Data sources

This study adhered to the READUS-PV guidelines to ensure transparent and standardized reporting of pharmacovigilance disproportionality analyses (26). Data were obtained from the U. S. Food and Drug Administration (FDA) Adverse Event Reporting System (FAERS), covering the period from the first quarter of 2014 to the first quarter of 2025. FAERS data consist of seven relational datasets in ASCII format, of which six were used in this study: demographic and administrative information (DEMO), drug information (DRUG), therapy start and end dates (THER), AEs coded using preferred terms (REAC), patient outcomes (OUTC), and indications for use (INDI) (27).

Infliximab-related reports were identified using both generic and brand names, including: “INFLIXIMAB,” “REMICADE,” “INFLECTRA,” “RENFLEXIS,” “ZYMFENTRA,” “CT-P13 SC,” “REMSIMA SC,” as well as synonyms such as “MONOCLONAL ANTIBODY CA2.” Drug roles in FAERS are categorized as primary suspect (PS), secondary suspect (SS), concomitant (C), and interacting (I). In this study, only reports in which infliximab was designated as the primary suspect (PS) drug were included to enhance signal specificity. AEs were coded using Preferred Terms (PTs) from the Medical Dictionary for Regulatory Activities (MedDRA, version 27.1).

### Data extraction and processing

#### Duplicate removal

Duplicate reports were identified and removed according to FDA recommendations. Specifically, reports sharing the same CASEID were considered duplicates, and only the most recent version was retained based on the FDA_DT (report date), ensuring that each case was uniquely represented (24).

### Case selection criteria

After deduplication, reports were included if they met the following criteria: (1) Infliximab was identified as the primary suspect (PS) drug; (2) The report contained at least one valid adverse event (PT); (3) The route of administration was available and classifiable.

### Handling of missing data

Reports with missing or ambiguous key variables—such as drug role, adverse event coding, or route of administration—were excluded from the analysis. No imputation was performed for missing data, in line with standard pharmacovigilance practices.

### Definition of exposure groups

The route of administration was determined based on the ROUTE field in the DRUG dataset. Reports were categorized into: (1) Intravenous (IV) administration; (2) Subcutaneous (SC) administration.

**Figure 1:**
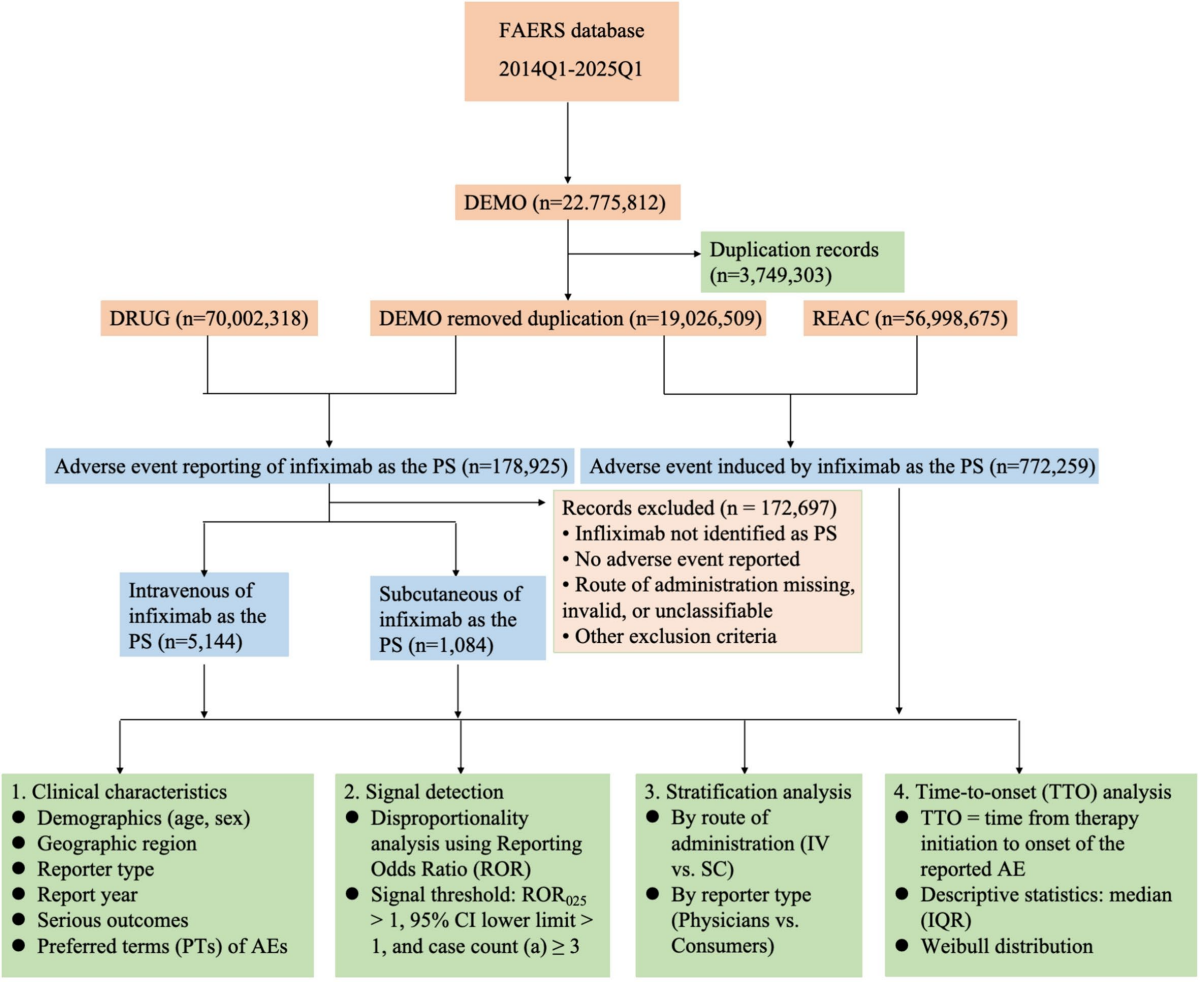
Flow chart of the study. FAERS, FDA Adverse Event Reporting System; DEMO, demographic and administrative information dataset; DRUG, drug information dataset; REAC, reaction/adverse event information dataset; THER, therapy dates dataset; OUTC, patient outcome dataset; IND, indication dataset; PS, primary suspect; PT, preferred term; IV, intravenous; SC, subcutaneous; ROR, reporting odds ratio; TTO, time-to-onset.

Standardized coding and manual verification were applied where necessary to ensure accurate classification. Reports with unclear or unspecified routes were excluded. Following data cleaning and screening, a total of 190,265,509 initial records were processed. Among these, 178,925 adverse event reports related to infliximab were identified. After applying inclusion and exclusion criteria, 5,144 reports were classified as intravenous administration and 1,084 reports as subcutaneous administration. A detailed flowchart of the data extraction and screening process is presented in Figure 1, illustrating the stepwise procedure from raw data acquisition to final analytical dataset construction.

### Statistical analysis

Disproportionality analysis was performed using the reporting odds ratio (ROR), a widely used pharmacovigilance method for signal detection in spontaneous reporting systems. This approach enables the identification of potential associations between drug exposure and AEs in the absence of denominator data. In this study, ROR was used to evaluate the association between subcutaneous and intravenous infliximab and the occurrence of AEs in the FAERS database (24). The corresponding two-by-two contingency table is presented in Supplementary Table 1. A signal was considered statistically significant when the lower limit of the 95% confidence interval (CI) of the ROR (ROR_025_) exceeded 1, with at least three reported cases. The formulas used to calculate the ROR and its 95% CI are provided in the Appendix (24).

Following signal detection, descriptive analyses were conducted to summarize the clinical characteristics of the reported cases, including patient demographics, reporter type, year of reporting, serious outcomes, and preferred terms (PTs) of AEs. Comparative analyses were further performed to evaluate differences in safety profiles between subcutaneous and intravenous infliximab. Time-to-onset (TTO) was defined as the interval between the initiation of infliximab therapy and the occurrence of the reported AE (24). TTO data were analyzed using medians, interquartile ranges (IQRs), and the Weibull distribution model to characterize the temporal pattern of AE occurrence.

The Weibull distribution is characterized by two parameters: the scale parameter (*α*), which reflects the dispersion of onset times, and the shape parameter (*β*), which describes changes in hazard over time. A *β* value < 1 with the upper limit of the 95% CI < 1 indicates a decreasing hazard over time (“early failure type”); a *β* value approximately equal to 1 with the 95% CI including 1 indicates a constant hazard (“random failure type”); and a *β* value > 1 with the lower limit of the 95% CI > 1 indicates an increasing hazard over time (“wear-out failure type”).

## Results

### Descriptive analysis

In the FAERS database, 178,925 adverse reaction events associated with infliximab were obtained from the first quarter of 2014 to the first quarter of 2025. After excluding 172,697 cases of unrelated records, 5,144 adverse reactions related to intravenous administration and 1,084 adverse reactions related to subcutaneous administration were included in the study. The clinical characteristics are detailed in Table 1. For intravenous administration, female showed a higher proportion of AEs (53.5%) compared to males (38.2%). In terms of age, middle-aged patients (18–64.9 years) exhibited the highest proportion (47.3%). Regarding adverse reaction outcomes, it is concentrated in other outcomes with 55.7%, followed by hospitalization outcomes at 34.4%. Concerning the country of the reporter, the majority focused primarily on Canada and US, separately accounting for 33.2 and 29.6%. In terms of reporter occupation, intravenous reports were mainly submitted by consumers (30.3%) and physicians (27.9%), followed by pharmacists (3.5%) and other health professionals (0.9%); reporter information was missing in 37.4% of cases.

**Table 1:** Clinical characteristics of patients with adverse events under different administration routes of infliximab.

Characteristics	Intravenous (*N* = 5,144)	Subcutaneous (*N* = 1,084)
Sex, *n* (%)		
Female	2,753 (53.5%)	488 (45.0%)
Male	1966 (38.2%)	307 (28.3%)
Missing	425 (8.3%)	289 (26.7%)
Age (year), *n* (%)		
<18 y	591 (11.5%)	86 (7.9%)
18 ~ 64.9 y	2,435 (47.3%)	403 (37.2%)
≥65 y	554 (10.8%)	112(10.3%)
Missing	1,564 (30.4%)	483 (44.6%)
Reported, *n* (%)		
Consumer	1,561 (30.3%)	320 (29.5%)
Health Professional	46 (0.9%)	44 (4.1%)
Pharmacist	179 (3.5%)	23 (2.1%)
Physician	1,433 (27.9%)	393 (36.3%)
Missing	1925 (37.4%)	304 (28.0%)
Outcome, *n* (%)		
Death	177 (3.4%)	49 (4.5%)
Disability	41 (0.8%)	20 (1.8%)
Hospitalization	1772 (34.4%)	387 (35.7%)
Life-Threatening	177 (3.4%)	41 (3.8%)
Other	2,977 (57.9%)	587 (54.2%)
Country, *n* (%)		
Canada	1708 (33.2%)	278 (25.6%)
Japan	228 (4.4%)	39 (3.6%)
United States	1,525 (29.6%)	457 (42.2%)
China	24 (0.5%)	2 (0.2%)
France	283 (5.5%)	76 (7.0%)
Other	1,376(26.75%)	232(21.4%)

For subcutaneous administration, female showed a higher proportion of AEs (45.0%) compared to males (28.3%). With respect to age, it is concentrated in the group of 18–64.9 years, accounting for 37.2%. In terms of adverse reaction outcomes, other outcomes exhibited the highest proportion with 42.6%, followed by hospitalization outcomes at 35.7%. Regarding the country of the reporter, US submitted the largest number of reports (42.2%) and Canada with 25.6%. For reporter occupation in the subcutaneous group, physicians accounted for the largest proportion (36.3%), followed by consumers (29.5%), other health professionals (4.1%) and pharmacists (2.1%); missing reporter information accounted for 28.0% of all subcutaneous cases.

### AE signals associated with different administration routes of infliximab

As shown in Table 2, it is observed that AEs vary across different administration routes. Under intravenous administration, the following adverse reactions showed more significant signal strengths: *Mycobacterium marinum* infection (*n* = 7, ROR = 100.42), tuberculous pleurisy (*n* = 11, ROR = 96.58), extrapulmonary tuberculosis (*n* = 5, ROR = 75.38), pulmonary tuberculosis (*n* = 72, ROR = 71), peritoneal tuberculosis (*n* = 7, ROR = 66.78), lymph node tuberculosis (*n* = 12, ROR = 66.03), disseminated tuberculosis (*n* = 37, ROR = 62.77), lupus-like syndrome (*n* = 123, ROR = 60.08), vaginal fistula (*n* = 4, ROR = 45.36), pouchitis (*n* = 8, ROR = 41.62), ileal perforation (*n* = 6, ROR = 38.7), tuberculosis (*n* = 110, ROR = 36.37), infusion related reaction (*n* = 520, ROR = 35.34), vulval abscess (*n* = 3, ROR = 3.08), polyserositis (*n* = 3, ROR = 31). Among them, vaginal fistula, pouchitis and polyserositis were identified as potential safety signals that are not currently described in the prescribing information. However, based on pathophysiological and clinical evidence, we propose that vaginal fistula and pouchitis are more likely attributable to disease progression, or to inadequate treatment response, than to direct drug-associated adverse effects. This observed data discrepancy may stem from consumers’ difficulty in distinguishing whether clinical manifestations are due to the natural course of the disease or are drug-associated, which may also reflect reporting biases in consumer-submitted data.

**Table 2:** Comparison of AE signal intensity under different administration routes.

Administration routes	PT	*N*	ROR (95%Cl)
Intravenous	*Mycobacterium marinum* infection	7	100.42 (47.41–212.72)
	Tuberculous pleurisy	11	96.58 (53.08–175.73)
	Extrapulmonary tuberculosis	5	75.38 (31.1–182.68)
	Pulmonary tuberculosis	72	71 (56.2–89.69)
	Peritoneal tuberculosis	7	66.78 (31.63–141.01)
	Lymph node tuberculosis	12	66.03 (37.31–116.85)
	Disseminated tuberculosis	37	62.77 (45.34–86.89)
	Lupus-like syndrome	123	60.08 (50.24–71.85)
	Vaginal fistula	4	45.36 (16.92–121.57)
	Pouchitis	8	41.62 (20.73–83.56)
	Ileal perforation	6	38.7 (17.31–86.51)
	Tuberculosis	110	36.37 (30.13–43.92)
	Infusion related reaction	520	35.34 (32.37–38.58)
	Vulval abscess	3	31.08 (9.98–96.82)
	Polyserositis	3	31 (9.95–96.56)
Subcutaneous	*Mycobacterium marinum* infection	5	179.1 (73.92–433.94)
	Blood iron abnormal	16	174.38 (106.29–286.09)
	Human antichimeric antibody positive	5	148.79 (61.49–360.01)
	Diabetic gastroparesis	3	81.24 (26.07–253.18)
	Nasal crusting	3	53.02 (17.04–164.96)
	Red blood cell sedimentation rate abnormal	9	39.04 (20.28–75.17)
	Juvenile idiopathic arthritis	7	26.57 (12.65–55.81)
	Papilloma viral infection	4	26.46 (9.91–70.61)
	Dysplasia	3	26.46 (8.52–82.2)
	Tenosynovitis	7	25.98 (12.37–54.57)
	Disseminated tuberculosis	6	25.21 (11.31–56.2)
	Blood urea decreased	3	24.41 (7.86–75.83)
	Sacroiliitis	4	22.11 (8.29–59.01)
	Alanine aminotransferase abnormal	4	21.1 (7.91–56.3)
	Dermatitis psoriasiform	4	20.91 (7.84–55.78)

PT, preferred term; N, number of cases with available; ROR, reporting odds ratio.

The signal intensity was markedly higher for these adverse reactions in subcutaneous administration: *Mycobacterium marinum* infection (*n* = 5, ROR = 179.1), blood iron abnormal (*n* = 16, ROR = 174.38), human antichimeric antibody positive (*n* = 5, ROR = 148.79), diabetic gastroparesis (*n* = 3, ROR = 81.24), nasal crusting (*n* = 3, ROR = 53.02), red blood cell sedimentation rate abnormal (*n* = 9, ROR = 39.04), juvenile idiopathic arthritis (*n* = 7, ROR = 26.57), papilloma viral infection (*n* = 4, ROR = 26.46), dysplasia (*n* = 3, ROR = 26.46), tenosynovitis (*n* = 7, ROR = 25.98), disseminated tuberculosis (*n* = 6, ROR = 25.21), blood urea decreased (*n* = 3, ROR = 24.41), sacroiliitis (*n* = 4, ROR = 22.11), alanine aminotransferase abnormal (*n* = 4, ROR = 21.1), dermatitis psoriasiform (*n* = 4, ROR = 20.91). We also identified 10 new adverse reactions not mentioned in package insert for drug, including blood iron abnormal, diabetic gastroparesis, nasal crusting, red blood cell sedimentation rate abnormal, juvenile idiopathic arthritis, papilloma viral infection, dysplasia, tenosynovitis, blood urea decreased, and sacroiliitis. Similarly, we tend to consider abnormal iron levels and decreased blood urea to be outcomes of disease progression rather than adverse drug reactions. Since these parameters are laboratory indicators associated with anemia, reporters may have included all abnormal values in the database without adequately distinguishing their underlying causes, thereby introducing a certain degree of data bias.

Our comparative analysis suggested that intravenous administration is more prone to adverse reactions related to infections such as *Mycobacterium marinum* infection, tuberculous pleurisy, extrapulmonary tuberculosis, pulmonary tuberculosis, peritoneal tuberculosis, lymph node tuberculosis, and disseminated tuberculosis when compared to subcutaneous injection. Additional, intravenous administration is also associated with higher reporting frequency adverse reactions, including infusion related reaction and lupus-like syndrome. The adverse reactions related to intravenous administration are almost all mentioned in the drug instructions. On the other hand, adverse reactions associated with infection and human antichimeric antibody positivity are frequently reported with subcutaneous administration, which might be a signal warranting further investigation. It is worth mentioning that subcutaneous administration is associated with AEs that are not listed in the product insert, including red blood cell sedimentation rate abnormal, juvenile idiopathic arthritis, tenosynovitis, and sacroiliitis.

### Distribution characteristics of common adverse reactions

**Figure 2:**
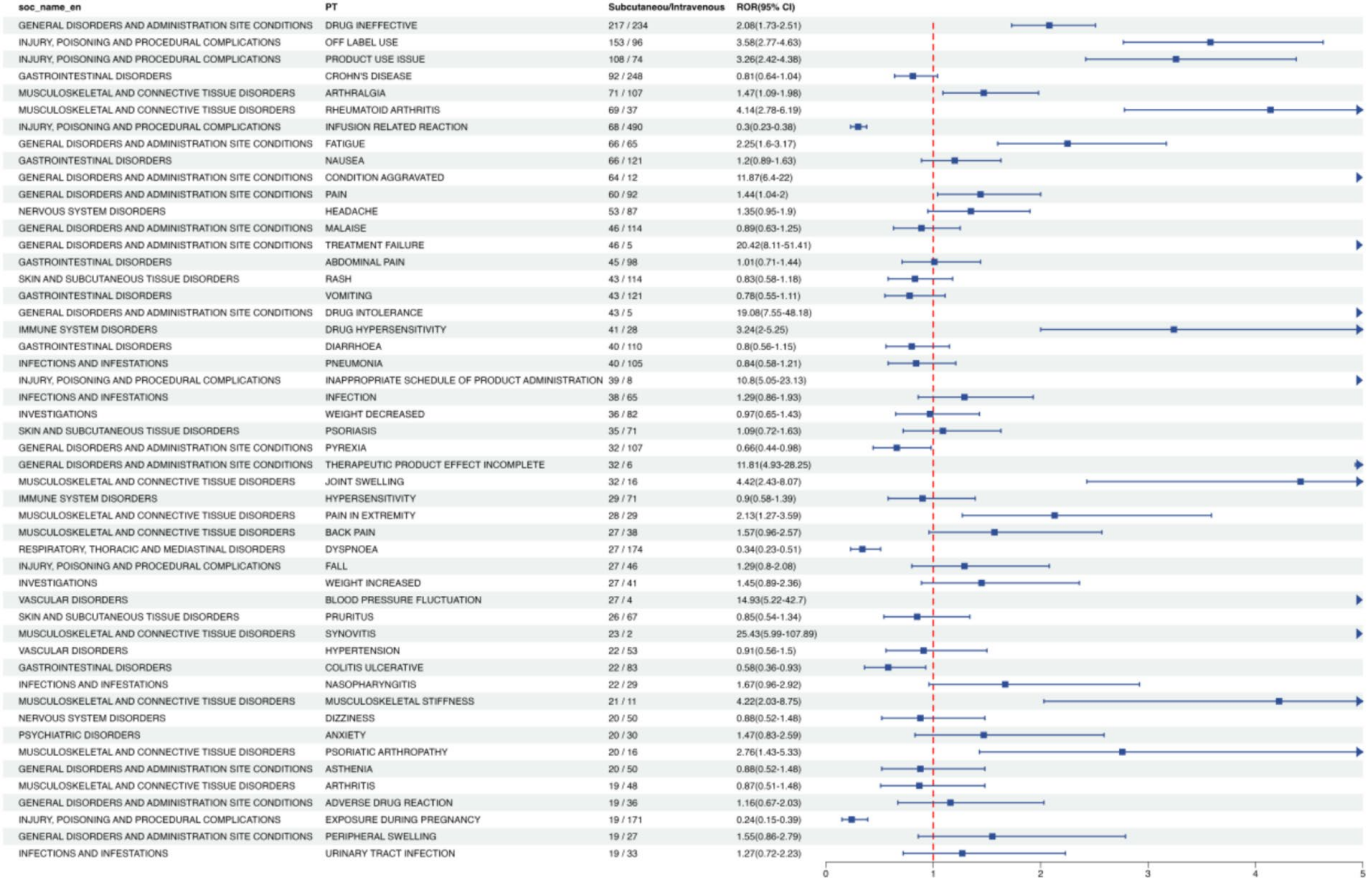
Analysis of differential risk signals for different administration routes of infliximab. Odds ratios (ROR) for the top 50 AEs are reported. PT, preferred term; SOC, system organ classes; ROR, reporting odds ratio. Adverse reactions were regarded as more likely to occur with subcutaneous administration if the ROR > 1 and the confidence interval did not include 1. On the contrary, adverse reactions were more likely to be associated in intravenous administration if the ROR < 1 and the confidence interval did not include 1.

In order to evaluate the impact of different administration routes on the common AEs associated with infliximab, we employed the ROR to identify AEs between intravenous and subcutaneous administration, and then ranked them based on ROR 95%CI values. We selected the top 50 AEs and classified them by system organ classes (SOC) for in-depth analysis. Figure 2 illustrates the comparative adverse event profiles associated with subcutaneous and intravenous administration of infliximab. Several adverse events demonstrated disproportionately higher reporting frequencies following subcutaneous administration, particularly musculoskeletal and connective tissue disorder-related events, including arthralgia, rheumatoid arthritis, joint swelling, back pain, pain in extremity, synovitis, and musculoskeletal stiffness. Signals related to drug intolerance and drug hypersensitivity were also more prominent in the subcutaneous group. Conversely, infusion related reaction and dyspnoea were more frequently reported following intravenous administration, consistent with the known safety characteristics of infusion-based therapy. Taken together, these findings suggest that administration route may influence the adverse event spectrum of infliximab. The observed differences in reporting patterns deserve further mechanistic and clinical investigation.

### Induction time of related adverse reactions under different administration routes of infliximab

**Figure 3:**
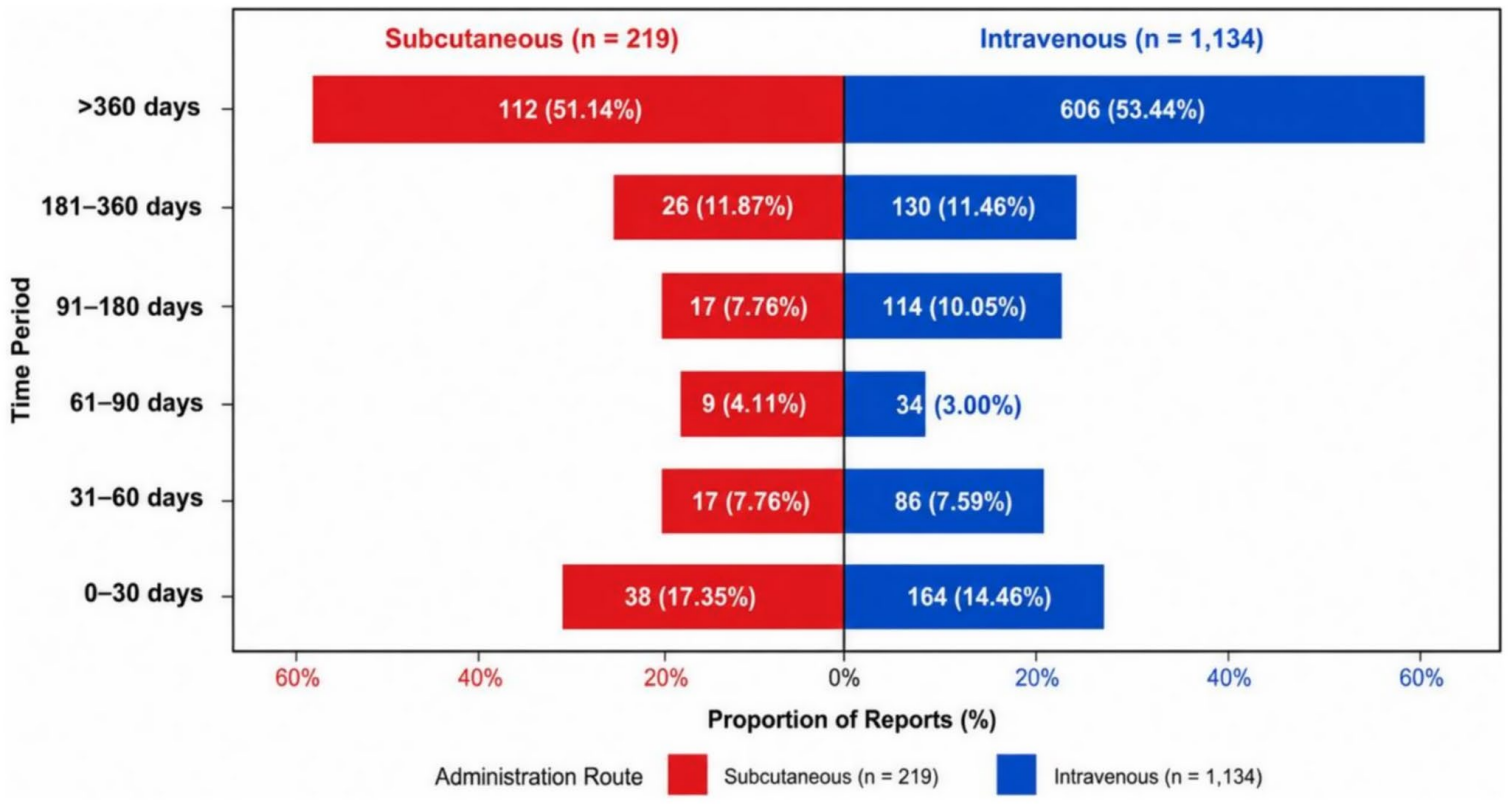
The induction time of adverse reactions associated under different administration routes of infliximab.

The results of the induction time of adverse reactions with different administration methods of infliximab, retrieved from the FAERS database, are presented in Figure 3. From the Figure 3, the incidence of adverse reactions to intravenous infliximab was 14.46% during 0–30 days, 7.58% during 31–60 days, 3.00% during 61–90 days, 10.05% during 91–180 days, 11.46% during 181–360 days, 53.44% over 360 days. Additionally, the incidence of adverse reactions to subcutaneous infliximab was 17.35% during 0–30 days, 7.76% during 31–60 days, 4.11% during 61–90 days, 7.76% during 91–180 days, 11.87% during 181–360 days, 51.14% over 360 days. Across all corresponding time periods, the incidence of adverse reactions with intravenous infliximab was consistently higher than that with subcutaneous infliximab. It is worth noting that the majority induction time of related adverse reactions for infliximab is over 360 days use, regardless of the administration method. One possible explanation is the accumulation of immunogenicity, amplification of opportunistic infection mechanisms and alterations in pharmacokinetics associated with infliximab, or other factors. AEs stratified by administration route (intravenous/subcutaneous) and reporter type (consumers/physicians) were ranked by ROR (top 10), which is shown in Table 3. For intravenous administration (consumers: 1561, 30.3%; physicians: 1433, 27.9%), consumers mainly reported intestinal resection and infusion related reaction, while physicians focused on *Mycobacterium marinum* infection and tuberculosis-associated AEs. For subcutaneous administration (consumers: 320, 29.5%; physicians: 393, 36.3%), consumer reports were dominated by antibody-positive events and immune-inflammatory disorders, whereas physician reports featured diabetic gastroparesis, blood iron abnormal and tuberculosis-related AEs, with notable differences in the spectrum of top AEs between the two reporter groups across both routes.

**Table 3:** Top 10 AEs (by ROR) in consumer vs. physician reports: intravenous and subcutaneous routes.

Intravenous	Consumers (1,561, 30.3%)			Physicians (1,433, 27.9%)		
	PT	ROR	*N*	PT	ROR	*N*
AEs (top 10 by ROR)	Intestinal resection	113.81	47	*Mycobacterium marinum* infection	242.23	4
	Post procedural discharge	71.47	4	Tuberculous pleurisy	147.51	4
	Prolonged labor	58.34	3	Peritoneal tuberculosis	120.93	3
	Lupus-like syndrome	56.3	40	Lymph node tuberculosis	116.26	5
	Coccidioidomycosis	29.55	3	Disseminated tuberculosis	114.95	16
	Pyoderma gangrenosum	29.14	8	Pulmonary tuberculosis	78.9	19
	Intestinal ulcer	22.98	3	Lupus-like syndrome	65.98	32
	Tuberculosis	22.89	24	Tuberculosis	49.18	35
	Infusion related reaction	22.72	117	Papillary thyroid cancer	31.59	3
	Anal fistula	15.93	7	Infusion related reaction	30.96	108

Subcutaneous	Consumers (320, 29.5%)			Physicians (393,36.3%)		
	PT	ROR	*N*	PT	ROR	*N*
AEs (top 10 by ROR)	Human antichimeric antibody positive	271.64	3	Diabetic gastroparesis	303.14	3
	Drug specific antibody	117.33	5	Blood iron abnormal	281.91	7
	Drug specific antibody present	35.07	7	Drug specific antibody	85.55	3
	Drug specific antibody	117.33	5	Red blood cell sedimentation rate abnormal	80.8	5
	Polyarthritis	18.97	3	Papilloma viral infection	73.97	3
	Red blood cell sedimentation rate increased	14.34	5	Sacroiliitis	61.84	3
	Blood iron decreased	11.4	4	Disseminated tuberculosis	46.95	3
	Synovitis	10.89	6	Juvenile idiopathic arthritis	42.39	3
	Ankylosing spondylitis	10.52	4	Tuberculosis	24.51	8
	Rheumatoid arthritis	6.65	23	Drug specific antibody present	18.26	3

PT, preferred term; ROR, reporting odds ratio; N, number of reports.

The top 10 AEs are ranked by ROR within each subgroup. Only signals meeting the predefined disproportionality criteria (ROR > 1 and 95% confidence interval not including 1) are presented.

### Time-to-onset analysis

The onset time results and weighted signal proportion analysis for clinically significant signals of infliximab AEs related to subcutaneous and intravenous administration are shown in Table 4. It’s demonstrated that the median onset time of infliximab AEs was 436.5 days (interquartile range [IQR]: 88–1,226) for intravenous administration and 376 days (IQR: 59.5–1791.5) for subcutaneous administration. From Table 4, we can observe that both the shape parameter *β* and the upper limit of the 95%CI are <1 in the Weibull distribution results for subcutaneous and intravenous administration, classifying this as an early failure type, which indicated that the incidence of AEs shows a declining trend with time.

**Table 4:** Time-to-onset analysis for signals with intravenous and subcutaneous prioritization.

Prioritization				Weilbull distribution				Failure type
Administrationroutes	Case	TTO (days)		Scale parameter		Shape parameter		
	*N*	Median (IQR)	Min-max	*α*	95%CI	*β*	95%CI	
Intravenous	1,134	436.5(88–1,226)	1–4,722	663.67	605.53–721.81	0.70	0.66–0.73	Early failure
Subcutaneous	219	376(59.5–1791.5)	1–5,234	748.03	577.45–918.61	0.61	0.54–0.68	Early failure

N, number of cases with available time-to-onset; IQR, interquartile range; TTO, Time-to-onset.

A *β* value < 1 with the upper limit of the 95% CI < 1 indicates a decreasing hazard over time (“early failure type”); a *β* value approximately equal to 1 with the 95% CI including 1 indicates a constant hazard (“random failure type”); and a *β* value > 1 with the lower limit of the 95% CI > 1 indicates an increasing hazard over time (“wear-out failure type”).

## Discussion

This study provides a comprehensive pharmacovigilance assessment of infliximab-associated adverse events across different routes of administration using real-world FAERS data. Our findings demonstrate that both the spectrum and time-to-onset of AEs differ between intravenous and subcutaneous infliximab, highlighting the importance of route-specific safety evaluation. Demographic patterns should be interpreted cautiously given the substantial proportion of missing data, but overall the findings support the need for route-specific safety evaluation.

These findings align with previous research and offer novel insights into differences in AEs related to the different administration routes of infliximab. Infection-related adverse reactions, *Mycobacterium marinum* infection, tuberculous pleurisy, extrapulmonary tuberculosis, pulmonary tuberculosis, peritoneal tuberculosis, lymph node tuberculosis, disseminated tuberculosis, and papilloma viral infection, are more frequently observed in different administration routes of infliximab, which are consistent with the black warning in the prescribing information. In addition, studies have shown that the increased risk with infection for infliximab was observed (28, 29) and recommended that medical evaluation for tuberculosis, HBV and opportunistic infections should be carried out prior to infliximab therapy (30). It’s well known that infliximab could block inflammatory pathways and exert significant immunomodulatory effects. A study indicate that the immune system of some patients treated with infliximab is compromised (31). Based on this evidence, patients treated with infliximab exhibit a reduced capacity to resist pathogenic bacteria, which may be the primary reason for the increased risk of infection associated with infliximab therapy.

Infusion-related adverse reactions are unique AEs associated with intravenous administration. Researches have revealed that most acute infusion-related adverse reactions are mild (not interfering with daily activities) or transient. For example, Shergy et al. (32) and Parigi et al. (33) demonstrated that approximately 10% of patients treated with infliximab experience acute infusion-related reactions, with the most common manifestations being headache, pruritus, and urticaria. Schaible (34) indicated that less than 2% of patients discontinue infliximab treatment due to infusion-related serious reactions. According to the package insert, patients could receive pre-treatment with antihistamines, hydrocortisone, and/or acetaminophen, and then along with reducing the infusion rate, especially for patients with a prior history of infusion-related reactions, to reduce the incidence of acute infusion-related adverse reactions. And in intravenous administration, a common adverse reaction such as dyspnea may be a manifestation of acute infusion-related reactions.

A research has indicated that the ADA rate in the subcutaneous administration group was lower than that in the intravenous administration group (35). In contrast, our findings indicate that reports of human anti-chimeric antibody (HACA) positivity in FAERS database are predominantly associated with subcutaneous administration. This observed association represents a safety signal that warrants further investigation. It highlights the need for confirmatory studies with definitive denominators to assess whether the subcutaneous route truly confers a higher immunogenicity risk. Evidence for infliximab immunogenicity emerged mainly during initial single doses, intermittent therapy, or upon re-administration after a prolonged break (15, 36). The main manifestations of delayed hypersensitivity reactions include myalgia, arthralgia, rash, polyarthritis, and fever, typically occurring 3–14 days after infliximab infusion (30). It is common knowledge that delayed hypersensitivity reactions are associated with immunogenicity. The data in this study indicated that the adverse reactions such as arthralgia, drug hypersensitivity, joint swelling, pain in extremity, are frequently observed in subcutaneous administration. Similarly, Buisson et al. (21) indicated that a patient developed non-specific myalgia following subcutaneous injection of infliximab. Our findings raise the possibility that musculoskeletal and connective tissue disorders-related adverse reactions, more likely to occur in subcutaneous administration, may be manifestations of delayed hypersensitivity reactions. We hypothesize that HACA binds to infliximab, forming immune complexes that deposit in tissues such as the joints, which subsequently leads to musculoskeletal symptoms like arthralgia. This hypothesis warrants further experimental validation.

Although Weibull analysis suggested an early failure pattern (*β* < 1), indicating a higher hazard in the early treatment phase, a substantial proportion of events occurred after prolonged treatment. This apparent discrepancy reflects the heterogeneous nature of FAERS data and suggests that both early-onset and delayed adverse events may coexist in clinical practice. From a clinical perspective, these findings have important implications for treatment selection and patient management in IBD. Current clinical guidelines recommend infliximab as a key biologic therapy for moderate-to-severe IBD, with both intravenous and subcutaneous formulations available in clinical practice (37, 38).

Our findings suggest that the choice of administration route should not only consider efficacy but also route-specific safety profiles. Intravenous administration is more commonly associated with infusion-related reactions and infections, which may require closer monitoring during administration. In contrast, subcutaneous administration offers greater convenience and may improve patient adherence and long-term treatment persistence, but clinicians should be aware of potential immunogenicity-related and musculoskeletal adverse events.

These differences are particularly relevant in individualized treatment strategies. For patients with a history of infusion reactions or those requiring more convenient treatment options, subcutaneous administration may be preferable. Conversely, for patients requiring closer supervision or with concerns about immunogenicity, intravenous administration may remain the preferred option.

Overall, integrating route-specific safety data into clinical decision-making may help optimize treatment selection, improve patient adherence, and enhance long-term disease management in IBD.

### Limitations

Several limitations inherent to FAERS-based analyses should be acknowledged. First, FAERS is a spontaneous reporting system subject to reporting bias, underreporting, and incomplete data, which may affect the reliability and representativeness of the findings. In particular, disproportionality signals derived from rare adverse event reports with small sample sizes may be unstable and should therefore be interpreted cautiously. Second, disproportionality analysis identifies statistical associations rather than causal relationships. Therefore, the observed signals should be interpreted cautiously, as they may reflect reporting tendencies rather than true risk differences. Third, potential confounding factors, including comorbidities, concomitant medications, and disease severity, cannot be adequately controlled in the FAERS database. These factors may influence both the occurrence and reporting of adverse events, thereby limiting causal inference. Fourth, the absence of denominator data precludes the estimation of incidence rates and absolute risk, restricting the ability to quantify the true magnitude of adverse events across different administration routes. Given these limitations, our findings should be considered hypothesis-generating. Future studies, including well-designed prospective cohort studies, real-world comparative studies with defined denominators, and mechanistic investigations into immunogenicity and delayed hypersensitivity reactions, are warranted to validate and further elucidate these observations.

## Conclusion

In summary, infliximab exerts its important role in controlling the inflammatory response in Crohn’s disease and ulcerative colitis by targeting and inhibiting TNF-*α*, which has led to its widespread therapeutic application. And clinicians should be aware of potential differences in reported adverse event profiles across administration routes, when selecting dosage forms. Provide individualized recommendations to patients based on their clinical status, comorbidities, and drug properties to minimize adverse reaction risks and optimize therapeutic outcomes.

## Data Availability

The datasets presented in this study can be found in online repositories. The names of the repository/repositories and accession number(s) can be found in the article/Supplementary material.
