# Supplementary material for: Safety evaluation of subcutaneous and intravenous administration of infliximab: a real-world study based on the FAERS database
Source: Front Med (Lausanne). 2026 Jun 1;13:1834815. doi: 10.3389/fmed.2026.1834815 (PMC13266594; doi:10.3389/fmed.2026.1834815)
Supplement: Supplementary file 1 [file Table_1.docx]

**Supplementary Table 1 The two-by-two contingency table**

| linked tables in chapters | | | |
| --- | --- | --- | --- |
|  | Target adverse drug event | Other adverse drug events | Sums |
| Infiximab | a | b | a+b |
| Other drugs | c | d | c+d |
| Sums | a+c | b+d | a+b+c+d |
| linked tables in chapters | | | |
|  | Target adverse drug event with infiximab | Other adverse drug events with infiximab | Sums |
| Subcutaneous | a | b | a+b |
| Intravenous | c | d | c+d |
| Sums | a+c | b+d | a+b+c+d |
